# The Effects of Health Coverage Schemes on Length of Stay and Preventable Hospitalization in Seoul

**DOI:** 10.3390/ijerph15040772

**Published:** 2018-04-17

**Authors:** Jungah Kim, Changwoo Shon

**Affiliations:** The Seoul Institute, 57 Nambusunhwan-ro, 340-gil, Seocho-gu, Seoul 06756, Korea; 0826kja@naver.com

**Keywords:** medical aid, preventable hospitalization, avoidable admission, length of stay, medical security

## Abstract

The Medical Aid program is government’s medical benefit program to secure the minimum livelihood and medical services for low-income Korean households. In Seoul, the number of Medical Aid beneficiaries has grown, driving an increases in the length of stay (LOS) and healthcare cost. Until now, studies have focused on quantity indicators, such as LOS, but only a few studies have been conducted on the service quality. We investigated both LOS and the preventable hospitalization (PH) rate as proxy indicators for the quantity and quality of services provided to Medical Aid beneficiaries in Seoul. To understand the program’s impact, we extracted appropriate data of Medical Aid beneficiaries and data of the lower 20% of National Health Insurance (NHI) enrollees, performed Propensity Score Matching (PSM), and controlled the variables related to disease severity. The differences between Medical Aid beneficiaries and NHI enrollees were estimated using multilevel analysis. The LOS of Medical Aid beneficiaries was longer, and the preventable hospitalization (PH) rate was higher than that of NHI enrollees. It implies that these beneficiaries did not receive timely and adequate healthcare services, despite their high rate of service utilization. Thus, indicators such as patient’s visits and screening related to PHs should be included in management policies to improve primary care.

## 1. Introduction

From 2007 to 2015, the number of Medical Aid beneficiaries in Korea has decreased from 1853 thousand people to 1534 thousand [1]. However, in Seoul, the capital city of Korea, the number of beneficiaries has increased from 239,760 to 244,363 [2]. With the increase in the number of beneficiaries, the total length of stay (LOS) has increased from 3,163,378 to 4,298,811 days, and the healthcare cost has also surged from $222,191.79 to $386,941.51, according to data on inpatients [2]. The health security rate in Korea is now 63.4% [2], due to gaps in covered services, and therefore, the lower social class is at risk of receiving poor healthcare services, despite the general availability of equal services to all classes of citizens. Most studies regarding the Medical Aid Program in Korea have focused on the quantities of healthcare utilization, such as LOS and healthcare cost [3,4,5], and only a few studies have investigated the quality of healthcare services [6,7]. The high healthcare utilization may be the result of high disease severity, and a low-income population segment is more likely to have severe diseases [8]. However, studies related to the high healthcare utilization have often interpreted the high cost as moral hazard or as “medical shopping” by Medical Aid beneficiaries who want to avoid out-of-pocket spending [9]. Prior studies used preventable hospitalization (PH) as an indicator in an effort to measure the quality of healthcare services [10,11,12]. The lack of timely and appropriate care may lead to illnesses that require hospitalization [12,13]. PH rates have also been used as an indicator to measure access to healthcare services [12]. Studies have shown that improved access to basic healthcare services decreases PH rates, and these PH rates are higher among vulnerable populations, such as lower social classes, minority racial/ethnic groups, and those without health insurance [6,11,12,13,14].

Against the backdrop of this situation, our study aims to investigate whether there are differences between Medical Aid and NHI patients in terms of LOS and PH rates, representative indicators for the quantity and quality of healthcare utilization. By extracting data on the lower 20% of the NHI enrollees based on insurance contribution, conducting Propensity Scores Matching (PSM) and controlling disease severity, our study aims to find out differences in LOS and PH rates derived from health coverage scheme differences, NHI and Medical Aid program.

## 2. Medical Aid Program in Korea

The Medical Aid program is government’s medical benefit program—a public assistance scheme to secure the minimum livelihood for low-income households and to assist self-help by providing medical services [2]. The Medical Aid program is primarily based on the National Basic Livelihood Security recipients. The beneficiaries are divided into Type 1 and 2 according to status, such as age, and whether he/she is able to work. Type 1 Medical Aid beneficiaries are those who are unable to work aged below 18 or over 65, and also includes intangible cultural assets, people who were injured or died while saving other people and property, and so on. Type 2 beneficiaries are those between the ages 18 and 65 who are able to work. In Seoul, the ratio of Type 1 to Type 2 beneficiaries is approximately 7 to 3. The two programs offer different medical out-of-pocket expense plans for outpatient/inpatient healthcare services at medical institutions. The out-of-pocket spending of Medical Aid beneficiaries is relatively low, from 0% to 15%, when compared to NHI enrollees whose out-of-pocket spending is officially 20% for inpatient healthcare services.

## 3. Materials and Methods

### 3.1. Design and Participants

Data on our sample of respondents were sourced from customized claims data from the National Health Insurance Service (NHIS) and data on environmental level variables from the Seoul Survey, Health Insurance Review and Association Service (HIRA). Customized claims data is nationally representative data, that includes claims data of all patients residing in Korea, and which can be used to estimate the economic status of patients based on each person’s health insurance contribution. Since this data is not sample data, but census data, which therefore ensures reliability. This study used customized claims data from 2013 to 2015, and extracted the Medical Aid beneficiaries and National Health Insurance (NHI) enrollees in Seoul. Specifically, we extracted data on the economically lower 20% of the NHI enrollees based on their health insurance contributions to compare the LOS and PH rates derived from health coverage schemes between the two groups, where Medical Aid beneficiaries are represented as low income and a vulnerable societal class. As explained above, the customized claims data is census data, which included the data of all patients who used healthcare services at least once. The data had to be cleaned for the purpose of our research. We excluded data of inpatients of long-term care hospitals that charge patients with case payment, which can result in differences in inpatients’ healthcare utilization and providers’ behavior. The unit of analysis in this research was based on disease episodes.

### 3.2. Measurements

#### 3.2.1. Outcome Variables

The LOS are the number of days for which the patients are hospitalized. In this study, we excluded the data whose LOS was recorded as “0” because it means that a patient was diagnosed with a different primary disease after he/she was diagnosed at first. Even though the patient was diagnosed with diseases related to PH at first, the LOS of “0” means he/she was diagnosed with the different diseases, which are not related to PH. Therefore, we excluded 6549 episodes, 8116 episodes, and 10,211 episodes for each year.

PH are those rates that might not have occurred had the patient received effective, timely, and continuous outpatient (ambulatory) medical care for certain chronic disease conditions [15]. According to prior studies on PH, we selected 6 diseases based on International Classification of Diseases (ICD)-10 codes, namely diabetes complications (E10-14), chronic obstructive pulmonary disease (J44), hypertension (I10), congestive heart failure (I20), bacterial pneumonia (I50), and angina (J15). We defined PH conditions as a group from 6 diseases based on primary and secondary diagnosis [6]. We coded the inpatients who were hospitalized due to 6 diseases as “1” and coded “0” for the remaining inpatients. PH rate was calculated as the ratio of episodes with 6 diseases among the total episodes from the data.

#### 3.2.2. Individual Variables: Level 1

The following individual level variables were included in Model 2, Model 3, and Model 4: sex (men, women), age (0–14, 15–44, 45–64, over 65), Diagnosis Related Group (DRG) severity (0, over 1), Charlson Comorbidity Index (CCI), and health coverage schemes (Medicaid Aid beneficiaries, lower 20% of the NHI enrollees). The DRG severity is a putative indicator classifying the severity level of disease from 0 to 3 by using the DRG Grouper software program in HIRA. A higher DRG severity score indicates a more severe level of disease. For PH analysis, we excluded the 0–14-year-old group in line with the definition of PH. The CCI is an evaluation method for disease severity that is obtained by conducting two steps: giving a weighted value to each of the 19 diseases, and revising the sum of weighted values. Generally, the sum of the weighted values is categorized as scores into “0, 1, 2, 3+” [16,17,18], and the severity of a patient’s disease can be evaluated from the scores, where a higher score means a more severe level of disease. The CCI includes myocardial infarction, congestive heart failure, peripheral vascular and cerebrovascular disease, dementia, chronic pulmonary disease (mild, severe-moderate), connective tissue disorders, gastric and gastrojejunal ulcers, diseases of the liver (mild), diabetes without complications, diabetes with end-organ, hemiplegie (paraplegia), renal disease (mild, moderate to severe insufficiency), non-metastatic cancer, leukemia, lymphoma, multiple myeloma, disease of the liver (moderate to severe), metastatic cancer, and Acquired Immune Deficiency Syndrome (AIDS).

#### 3.2.3. Medical Institution Variables: Level 2

The following medical institution level variables were included in Model 3 and Model 4: classification of medical institutions (general hospital, hospital, and clinic), the number of doctors, the specialist ratio, the number of nurses, the number of beds, Seoul Municipal Hospitals (whether hospitals are included in Seoul Municipal Hospitals or not), and the medical treatment year (2013, 2014 and 2015).

#### 3.2.4. Environmental Variables: Level 3

The following environmental level variables were included in Model 4: the number of doctors per 10,000 persons, the number of nurses per 10,000, the number of beds per 10,000, the number of Computed Tomography (CT) per 10,000, the number of Magnetic Resonance Imaging (MRI) per 10,000, the number of Positron Emission Tomography (PET) per 10,000, and the number of people older than 65 per 10,000. CT, MRI, and PET are represented as high medical equipment.

### 3.3. Statistical Analysis

#### 3.3.1. Propensity Score Matching (PSM)

Propensity Score Matching (PSM) is widely used in observational studies to reduce selection bias. Observational study lacks randomization, hence, statistical inferences without bias adjustments usually include observed or unobserved effects of covariates [19]. We performed PSM to estimate the effect of a policy, Medical Aid Program, by accounting for the covariates that predict receiving the treatment. We included the factors “sex”, “age”, and “residence” in PSM, and estimated the propensity scores for both NHI enrollees and Medical Aid beneficiaries. We matched one NHI enrollee to two Medical Aid beneficiaries (1:2 matching) by using propensity scores.

#### 3.3.2. Multilevel Regression Analysis

Multilevel regression was used to estimate the difference in LOS and PH rates according to health coverage schemes at the individual, medical institution, and environmental levels. In the multilevel regression model, there are three fit statistics, namely Akaike’s Information Criterion (AIC), Bayesian Information Criterion (BIC), and −2 Log Likelihood. These fit statistics judge relative suitability of regression models by using Likelihood Function. Among fit statistics, AIC chose the regression model which has the minimum information loss by reflecting the Likelihood Function and the number of parameters [20]. Therefore, a smaller AIC implies that a regression model minimizes its information loss. The following four models were run for each outcome: Model 1, the null model, did not contain any covariates so that individual, medical institution, and environmental level variance in the outcomes in the absence of any explanatory variables could be assessed. Model 2 contained only the individual level covariates. Model 3 contained the individual and medical institution level covariates, and Model 4 contained all the individual level covariates, medical institutions, and the environmental level covariates. A model for these estimation methods is explained in the following statement, where in $Y_{ij}$ is the LOS and PH rate, $X_{ij}$ is the individual (i)’s characteristics using healthcare services in j medical institution, and $Z_{j}$ is the environmental characteristics of the district where j medical institution is located.

(1) $$\left\{P_{r}\left(Y_{ij}=1|X_{ij},\;Z_{j}\right)\right\}=\;\gamma_{00}+\gamma_{10}X_{ij}+\gamma_{01}Z_{j}+\gamma_{11}X_{ij}Z_{J}+U_{1j}X_{ij}+U_{0J}+\varepsilon_{ij}$$

## 4. Results

### 4.1. Descriptive Statistics

The descriptive statistics of the study sample are provided in Table 1. In total, 323,946 episodes of Medical Aid beneficiaries and 591,069 episodes of the lower 20% of the NHI enrollees were included in the study. Men and women were similarly distributed in each group and year. When looking at age groups, the rate of the 45~64 year age group was the highest, and the rate of 0~14 year age group was the lowest. The rate of DRG severity 0 was higher than DRG severity over 1. When looking at the classification of medical institutions, over half of the respondents among both Medical Aid beneficiaries and the lower 20% of NHI enrollees were hospitalized in general hospitals for over 3 years. The hospitalization rate of Medical Aid beneficiaries in hospitals and clinics were about 31% and 6%. Compared to Medical Aid beneficiaries, more NHI inpatients were hospitalized in hospitals. The rate of Medical Aid inpatients hospitalized in Seoul metropolitan hospitals was approximately 12%, and this rate was four times higher than the lower 20% of the NHI inpatients. For healthcare utilization, the average LOS and PH rates of Medical Aid beneficiaries were higher than the lower 20% of the NHI enrollees.

### 4.2. Multilevel Analysis

Individual and environmental factors associated with LOS are shown in Table 2. Model 2 shows the correlation between the average LOS and individual factors including sex, age, the severity of diseases, such as DRG severity and CCI, and the health coverage scheme. When looking at individual factors, the average LOS of women was significantly shorter than that of men. Furthermore, the 15–44, 45–64, and the over 65 years old age groups had significantly longer stays than the 0–14 age group. When looking at the severity of diseases, the DRG severity and CCI were associated with the length of stay. In other words, the more severe disease was, the longer inpatients were hospitalized. The health coverage scheme was associated with the LOS where Medical Aid beneficiaries’ LOS was longer than the lower 20% of the NHI enrollees. In Model 3, we added the medical institution level predictors. When looking at medical institutions, the inpatients in general hospitals stayed shorter than those in clinics; however, the inpatients in hospitals stayed longer than those in clinics. At institutions where the ratio of doctors and nurses to patients is higher, the LOS was significantly lower; however, the number of beds in medical institutions was negatively associated with the length of stay. The ratio of medical specialist was related to the LOS. Specifically, when the rate of medical specialist increases, about 0.3 days of LOS was decreased. In addition, the LOS of inpatients in Seoul metropolitan hospitals was longer than stays in medical institutions that were not included in Seoul metropolitan hospitals. The medical treatment year had an associations with the length of stay for 2015 compared to 2013. Model 4 shows the associations between the LOS and predictors from individual to environment level. The results show that the LOS was not affected by environmental factors.

The associations between PH rate and predictors from an individual and environmental level are shown in Table 3. Model 2 shows the correlation between PH and individual factors. The figure for women’s PH was significantly lower than that of men. When looking at age groups, the likelihood of PH of the 45–64 and the over 65 age group was higher than that of the 15–44 age group. The DRG severity and CCI also showed association PH. The inpatients with severe diseases are more likely to be hospitalized due to preventable diseases. The health coverage scheme was significantly associated with preventable hospitalizations, which means that the Medical Aid beneficiaries’ PH rate was higher than that of the lower 20% of the NHI enrollees. In Model 3, we included the medical institution level predictors. When considering the medical institutions, the PH rate of inpatients in general hospitals and hospitals was higher than that of the clinics. At institutions where the number of doctors to patients was higher, the PH rate was significantly lower; however, the coefficient was small. The ratio of medical specialist was also related to PH rates where the rate of medical specialist increases, about 4.8% of PH rate was decreased. The number of nurses and beds were not associated with the PH rates. For Seoul metropolitan hospitals, the PH rate of inpatients in Seoul metropolitan hospitals was relatively higher than that of inpatients not included in Seoul metropolitan hospitals. The medical treatment year had associations with the length of stay for 2014 compared to 2013. Model 4 shows the associations between the PH rates and predictors from individual to an environmental level. The results show that the PH rates was not affected by environmental factors same as LOS.

## 5. Discussion

The main purpose of this study is to investigate the differences between the Medical Aid beneficiaries and the lower 20% of the NHI enrollees in terms of both quantity and quality of healthcare utilization. Through PSM, and by controlling covariates in the regression model, we explored the difference in LOS and PH rates derived from differences in health coverage scheme benefits. We used LOS as a proxy indicator for the quantity of healthcare where LOS refres to the number of days that inpatients spend in hospital. In addition, the PH rate was used as a putative indicator for the quality of healthcare where it indicates avoidable admissions if only patients had received timely and appropriate care. Prior studies have used these two indicators [3,4,5,10,11,12] to explore healthcare services, and therefore, we also applied LOS and PH rate in our regression model.

First, concerning LOS, Medical Aid patients had significantly longer stays than that of NHI patients, and this result is consistent with prior studies [21,22,23,24]. The Medical Aid patients are more likely to use inpatient medical services because of the low out-of-pocket spending, and this was previously regarded as the “moral hazard” of Medical Aid patients [8]. Considering that the rate of Medical Aid inpatients who decided hospitalization on their own was 12.1%, which was higher than the lower 20% of the NHI enrollees (8.6%) and the total NHI enrollees (10.1%) [25], Medical Aid patients can decide their healthcare utilization freely, so they tend to use inpatient medical services. To reduce this excessive healthcare utilization, comprehensive measures for the Medical Aid program were established in 2006 by introducing a partial out-of-pocket cost to beneficiaries. Kim et al. noted that the number of Medical Aid inpatients increased after the introduction of measures to charge a co-payment for outpatient services [23]. Thus, we can infer that there is a gap in LOS between Medical Aid beneficiaries and the lower 20% of the NHI enrollees due to a difference in out-of-pocket spending, despite adjusting individual and clinical characteristics. Concerning the patient-side, there is a provider-side effect that providers increase the quantity of healthcare services because they do not have to consider the out-of-pocket spending of patients. Joo et al. showed that the intensity of providers’ medical treatment is stronger for Medical Aid patients, who do not pay or those who indulge in less out-of-pocket spending, than the NHI enrollees [26].

Second, the PH rate of Medical Aid patients was higher than that of the NHI patients, and it was statistically significant. This is due to a lack of timely, appropriate, and adequate primary care services, which could have curbed PH [12,27,28]. From the perspective of the phased health system and medical institutions, primary care is the first phase pertaining to medical institutions, such as clinics and hospitals, which provide inpatient and outpatient healthcare services [29]. According to Organization for Economic Cooperation and Development (OECD) statistics, the per capita doctors’ consultations in Korea were 16.0, the highest among OECD countries [30]. Furthermore, the physician visits per one person, the representative variable for access to primary care, of Medical Aid beneficiaries in Seoul, are 31.1 visits [31]. In light of these two statistics, the quality and appropriateness of healthcare services might be insufficient, because the PH rate was high, in spite of many doctors’ consultations and physician visits. Moreover, Medical Aid beneficiaries fail to manage their health behaviors, such as drinking and walking [25], which result in PHs.

This study examined the differences of LOS and PH rates between Medical Aid and NHI patients, notwithstanding some limitations. There is a limitation in the data source. In Korea, there is no data which encompasses both claims and behavioral variables. The National Health Insurance claims data has the advantage of including all patients who live in Korea’s claims data, although it does not include health behavioral variables. We extracted the lower 20% of NHI patients—the low-income group among NHI enrollees—and compared this data with that of the Medical Aid patients, based on similar income trends for the two groups. Studies show that the low-income population is likely to indulge in unhealthy behavior, such as smoking, drinking, and insufficient exercise [32,33,34]. Thus, we can minimize the behavioral differences by the assumption that health behaviors between the two groups are similar based on their similar income.

## 6. Conclusions

This study reviewed both the quantity and quality of healthcare utilization of Medical Aid patients. Using PSM and controlling variants, this study specifically tried to highlight the differences between the quantity and quality of the medical care received by the Medical Aid patients compared to the lower 20% of NHI patients. The differences between the two groups are a result of the differences in the health coverage scheme, despite the fact that both groups being in similar situations. The LOS of Medical Aid patients was longer and the PH rate was higher than that of the lower 20% of NHI patients. This result might be caused by differences in out-of-pocket spending from patients’ and providers’ sides, the appropriateness of primary care services, and health behavior management. Therefore, policies to decrease the LOS and PHs are needed. First, we can consider reforming the payment system to decrease the LOS. In the past, to reduce excessive healthcare utilization, policies charging the out-of-pocket spending to Medical Aid patients have been delivered. However, it is hard to decrease the quantity only with patient-side policy, reforming the payment system will be needed from provider-side. In addition, it is essential to formulate policies to improve the quality of primary care services are needed, such as managing indicators like patient’s visits, prescription, and screening related to PHs. In addition, the healthcare delivery system—including primary care—should be reconstructed to reduce PHs. Moreover, health promotion policies to manage health behaviors of Medical Aid beneficiaries, such as drinking, walking, and exercising, are necessary. This study examined the overall healthcare of Medical Aid patients, and future research should investigate specific diseases such as mental illnesses to develop appropriate target policies.

## Figures and Tables

**Table 1:** Descriptive characteristics of study sample, length of stay, and preventable hospitalization.

Classification	Variable		2013		2014		2015	
			Medical Aid (*n* = 106,424)	National Health Insurance (NHI) 20% (*n* = 186,736)	Medical Aid (*n* = 109,980)	NHI 20% (*n* = 196,099)	Medical Aid (*n* = 107,542)	NHI 20% (*n* = 208,234)
Individual-level	Sex	Men (%)	55.5	46.9	55.4	48.0	47.9	55.1
		Women (%)	44.5	53.2	44.7	52.0	52.2	44.9
	Age group	0~14 years old (%)	7.3	4.9	7.7	5.2	6.6	4.9
		15~44 years old (%)	21.5	23.7	19.9	22.3	19.3	21.0
		45~64 years old (%)	52.2	56.1	52.7	56.5	53.2	56.2
		Over 65 years old (%)	19.1	15.4	19.6	15.9	21.0	17.9
	DRG severity	DRG severity 0 (%)	69.2	59.0	68.6	59.9	67.3	57.5
		DRG severity over 1 (%)	30.8	41.0	31.4	40.1	32.7	42.5
Medical institution-level	Classification of medical institution	General Hospital (%)	62.3	60.8	62.4	61.2	62.6	61.1
		Hospital (%)	31.5	24.1	31.6	24.5	32.1	25.1
		Clinic (%)	6.2	15.1	6.0	14.3	5.3	13.8
	Seoul Metropolitan Hospital (%)		12.3	3.5	13.0	3.4	12.3	3.2
Healthcare utilization	Average Length of stay (day)		24.3	8.8	23.7	8.7	24.0	8.6
	Preventable hospitalization (%)		5.8	3.2	6.1	3.4	6.0	3.5

**Table 2:** Multilevel analysis results—length of stay.

	Model 1			Model 2			Model 3			Model 4		
	Estimate	S.E	Pr > |t|	Estimate	S.E	Pr > |t|	Estimate	S.E	Pr > |t|	Estimate	S.E	Pr > |t|
Intercept	9.396	0.140	<0.0001	5.018	0.117	<0.0001	6.809	0.165	<0.0001	5.785	0.755	<0.0001
Individual-level predictors												
Sex (Men, reference)												
Women				−1.443	0.026	<0.0001	−1.369	0.026	<0.0001	−1.369	0.026	<0.0001
Age, groups (0–14, reference)												
15–44				3.571	0.057	<0.0001	2.460	0.058	<0.0001	2.459	0.058	<0.0001
45–64				4.713	0.053	<0.0001	3.498	0.054	<0.0001	3.497	0.054	<0.0001
Over 65				4.408	0.058	<0.0001	3.668	0.059	<0.0001	3.669	0.059	<0.0001
DRG Severity (o, reference)												
Over 1				2.787	0.029	<0.0001	2.811	0.029	<0.0001	2.811	0.029	<0.0001
Charlson Comorbidity Index (CCI)				−0.189	0.010	<0.0001	0.236	0.010	<0.0001	0.236	0.010	<0.0001
Medical Aid recipients (20% of NHI enrollees, reference)				4.825	0.027	<0.0001	4.387	0.028	<0.0001	4.387	0.028	<0.0001
Medical institution-level predictors												
Classification of Medical institutions (Clinics, reference)												
General hospitals							−2.456	0.064	<0.0001	−2.456	0.064	<0.0001
Hospitals							1.985	0.055	<0.0001	1.984	0.055	<0.0001
The number of doctors							−0.005	0.000	<0.0001	−0.005	0.000	<0.0001
The rate of medical specialist							−0.315	0.097	0.001	−0.311	0.097	0.001
The number of nurses							−0.002	0.000	<0.0001	−0.002	0.000	<0.0001
The number of beds							0.005	0.000	<0.0001	0.005	0.000	<0.0001
Inclusion in Seoul Metropolitan Hospitals (n/a, reference)							1.155	0.056	<0.0001	1.154	0.056	<0.0001
Medical treatment year (2013, reference)												
2014							−0.007	0.032	0.820	−0.043	0.051	0.394
2015							−0.146	0.032	<0.0001	−0.215	0.084	0.011
Environment-level predictors												
The rate of doctors										0.000	0.000	0.324
The rate of nurses										0.000	0.000	0.831
The rate of beds										−0.002	0.040	0.958
The rate of CTs										1.632	1.178	0.166
The rate of MRIs										−1.073	1.325	0.418
The rate of PETs										1.467	1.309	0.263
The rate of aged population over 65										0.074	0.066	0.263
Model Fit												
Akaike’s Information Criterion (AIC)	6,854,927			6,796,046			6,404,940			6,404,960		

Note: S.E, standard error; n/a, not applicable.

**Table 3:** Multilevel analysis results—preventable hospitalization.

	Model 1			Model 2			Model 3			Model 4		
	Estimate	S.E	Pr > |t|	Estimate	S.E	Pr > |t|	Estimate	S.E	Pr > |t|	Estimate	S.E	Pr > |t|
Intercept	−3.090	0.024	<0.0001	−3.589	0.032	<.0001	−4.728	0.071	<0.0001	−4.989	0.192	<0.0001
Individual-level predictors												
Sex (Men, reference)												
Women				−0.501	0.011	<0.0001	−0.472	0.012	<0.0001	−0.470	0.012	<0.0001
Age, groups (15–44, reference)												
45–64				0.822	0.018	<0.0001	0.804	0.018	<0.0001	0.802	0.018	<0.0001
Over 65				1.174	0.020	<0.0001	1.113	0.020	<0.0001	1.120	0.020	<0.0001
DRG Severity (o, reference)												
Over 1				0.369	0.012	<0.0001	0.225	0.012	<.0001	0.225	0.012	<0.0001
Charlson Comorbidity Index (CCI)				0.044	0.004	<0.0001	0.001	0.004	0.741	0.002	0.004	0.620
Medical Aid beneficiaries (20% of NHI enrollees, reference)				0.578	0.011	<0.0001	0.472	0.011	<0.0001	0.470	0.011	<0.0001
Medical institution-level predictors												
Classification of Medical institutions (Clinics, reference)												
General hospitals							1.909	0.055	<0.0001	1.909	0.055	<0.0001
Hospitals							1.469	0.054	<0.0001	1.466	0.054	<0.0001
The number of doctors							−0.001	0.000	<0.0001	−0.001	0.000	<0.0001
The rate of medical specialist							−0.482	0.037	<0.0001	−0.463	0.037	<0.0001
The number of nurses							0.000	0.000	<0.0001	0.000	0.000	<0.0001
The number of beds							0.000	0.000	<0.0001	0.000	0.000	<0.0001
Inclusion in Seoul Metropolitan Hospitals (n/a, reference)							0.091	0.019	<0.0001	0.090	0.019	<0.0001
Medical treatment year (2013, reference)												
2014							0.054	0.014	<0.0001	0.041	0.017	0.014
2015							−0.004	0.014	0.745	−0.028	0.023	0.223
Environment-level predictors												
The rate of doctors										0.000	0.000	0.936
The rate of nurses										0.000	0.000	0.534
The rate of beds										−0.004	0.009	0.657
The rate of CTs										0.294	0.262	0.261
The rate of MRIs										−0.215	0.302	0.476
The rate of PETs										−0.384	0.299	0.200
The rate of aged population over 65										0.018	0.016	0.248
Model Fit												
AIC	307,353			295,441			284,028			284,036		

Note: S.E, standard error; n/a, not applicable.
